# The Aesthetic Restoration of Anterior Tooth Fracture Using the Template Technique: A Case Report

**DOI:** 10.7759/cureus.68758

**Published:** 2024-09-05

**Authors:** Anjori A Raut, Punit Fulzele

**Affiliations:** 1 Pediatric Dentistry, Sharad Pawar Dental College, Datta Meghe Institute of Higher Education and Research, Wardha, IND

**Keywords:** aesthetic, anterior teeth, composite resin, fracture, restoration

## Abstract

Crown fractures are prevalent among children, and they cause major functional, aesthetic, and psychological issues. Clinicians must propose excellent aesthetics in the front section, as well as an explicit treatment strategy. Many cases require repeated reconstructions due to deteriorated findings over time. The most important goal among both children and their parents is to complete a promising repair that maintains its attractiveness and strength. This report presents a case of a permanent maxillary central incisor with an incisal crown fracture that was treated with composite resin repair.

## Introduction

Trauma to the anterior tooth is the most common type of dental injury in children and adolescents. There are two most common factors that lead to trauma: firstly, the trauma that occurs during sports - ball hitting the mouth, bicycle injuries, skateboard injuries, or extreme sports-related injuries; second, fracture of the tooth due to restoration or carious lesion which make the tooth fragile. Fractures of teeth may occur also due to road accidents, industrial accidents, bruxism, or sports injuries. Traumatic damage to permanent incisors occurs in 6-37% of cases globally. There are documented seasonal differences in the occurrence of trauma. Traumatic injuries to the tooth and surrounding periodontium require immediate diagnosis and treatment as it has both physiological and psychological impacts. This is crucial to prevent future complications in the emerging permanent teeth with insufficient root development [1].

Coronal restoration of anterior teeth is essential for aesthetics. Such a fracture may compromise aesthetics, function, tissue biology, and occlusal physiology, threatening tooth life and integrity. Coronal fractures of permanent incisors account for about 18-22% of all dental hard tissue trauma, with 28-44% constituting enamel and dentin crown fracture and 11-15% affecting the pulp. Coronal fractures caused by dental trauma most commonly affect maxillary front teeth, with mandibular fractures occurring less frequently. The sort of injury sustained is determined by the severity and direction of the stress, as well as the tissue's health and tolerance [2].

For anterior dental fractures, direct and indirect restorations are clinically effective therapeutic choices. There is no laboratory phase involved with direct restorations. They often combine adhesive methods, a variety of resin composites, and an acid-etching procedure for enamel and dentin. Indirect restorations need to be performed in a lab. Porcelain veneers and resin composites can be applied. Direct or indirect approaches can be employed for the restoration of a maxillary permanent incisor's fractured incisal edge. Direct restorations rely on the operator's expertise and experience as well as their adherence to a meticulous and problem-solving methodology. Carefully chosen opaquers, tints, and composite hues are used to repair teeth. An indirect restoration is created using an imprint outside of the mouth and bonded to the tooth afterward [3]. This report discusses a case of the aesthetic rehabilitation of damaged anterior teeth replaced with composite resin.

## Case presentation

**Figure 1:**
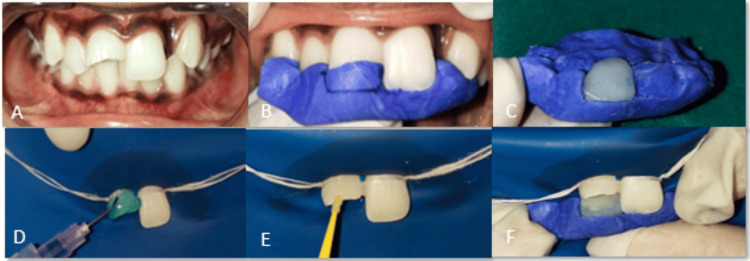
Restoration of the anterior tooth using the template technique

A 12-year-old female patient presented to the Department of Pediatric and Preventive Dentistry at Sawangi, Wardha, with a history of trauma due to a fall two years prior. Upon clinical examination, it was discovered that there was a Class 2 Ellis fracture pertaining to tooth #11, which did not cause any symptoms and did not result in any related soft or hard tissue damage to the supporting structures (Figure 1A). In radiographic examination, an intraoral periapical (IOPA) X-ray was advised and an electric pulp test (EPT) was done to check the vitality of the tooth. Clinical and radiographic evaluation demonstrated that the fracture involved the pulp. IOPA revealed an open apex with 11. A test for the vitality of the electric pulp indicated that the tooth was not vital. As a result, the Ellis Class 4 diagnosis was made. Apexification was done using mineral trioxide aggregate (MTA) to develop the apex and make subsequent treatment easier as the tooth's apex was open. After apexification, the canal was obturated 24 hours later using cold lateral compaction of gutta-percha (GP), and a sectional imprint of the upper anterior teeth was taken using putty (Zhermack S.p.A., Badia Polesine, Italy) (Figure 1B).

Using the neighboring central incisor as a guide, the anterior tooth 11 was carved on the template using a blade (Figure 1C). The template was then placed on the patient's teeth. As seen in Figure 1C, the palatal surface of the impression was constructed using enamel shade (E1) (Spectra ST Effects, Dentsply Sirona, Charlotte, NC), which was then cured for 20 seconds. A rubber dam was used to isolate the operating field, and 37% phosphoric acid was used to etch the tooth (Figure 1D). The lingual and facial surfaces were covered with adhesive (3M, St. Paul, MN), which was then cured (Figure 1E). The template was then placed on the palatal side (Figure 1F) and was attached and cured using dentin shade (D1) (Spectra ST Effects, Dentsply Sirona).

**Figure 2:**
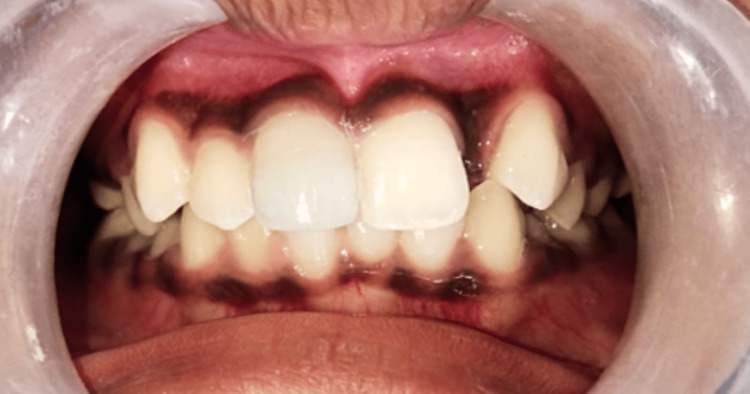
Final restoration after finishing and polishing

On the extended part of the composite, the facial aspect was formed. The lingual and incisal contours were determined, and dentin resin was applied to the middle third, allowing for the formation of dentinal lobes in the incisal area. The dentin mamelons were made of transparent resin (Spectra ST Effects, Dentsply Sirona), i.e., E1 and D1 were used between them. The enamel layer was placed on the facial area and distributed. After finishing and polishing, the final restoration was completed (Figure 2).

## Discussion

Fracture of a permanent incisor is a traumatic experience for young patients and causes significant psychological and social impacts on both parents and children. Malikaew et al. (2006) [4], Glendor (2008) [5], and Nagarajappa et al. (2020) [6] have highlighted how males are at a higher risk of suffering catastrophic tooth injuries since they are more likely to participate in extreme sports and adventure activities. Of note, 10.2% of children were found to have experienced anterior dental trauma; 64.3% of the 70 kids who experienced traumatic dental injury (TDI) were boys, and 35.7% were girls. Nonetheless, there was no significant correlation (p=0.109) between the gender traits of children exposed to anterior dental damage and those features. Central incisors were the teeth most afflicted, with maxillary anterior teeth damaged more often than mandibular teeth.

The maxillary and mandibular canines were the least affected teeth. Clinicians face significant challenges in managing patients with anterior tooth fractures, both in terms of function and aesthetics. Treatment goals may change based on the patient's age, intraoral state, and socioeconomic situation at the time of planning. Of note, 69% of cases involved enamel fracture, compared to 24% for pulpal damage or crown discoloration and 7% for dentin and enamel fracture. The majority of anterior dental trauma was seen in children older than five years. Dental injuries were more common in men than in women. The socioeconomic status (SES) of their parents was not substantially linked with anterior dental trauma; however, it was comparatively more common in high SES (57.2%) compared to low SES (42.9%) [7].

For the repair of broken teeth, a variety of treatment options are available, including fixed prostheses, composite restorations, and, if available, reattaching the fracture fragment before moving on to post and core-supported restorations. Once several re-bonding/composite resin restorations have been completed and this option is no longer practical, well-known treatment alternatives like laminated veneers or full-coverage restorations may be considered. Additionally, they often compromise the healthy tooth structure, making it difficult for the dentist to match the neighboring unrestored teeth. Direct composite restorations involve the direct application and shaping of composite resin onto the prepared tooth surface within a single dental appointment. This approach is particularly suitable when the remaining tooth structure can adequately support the restoration and when immediate chairside adjustments are advantageous. In the case of the Ellis Class 4 fracture, where the tooth had undergone apexification and root canal treatment, direct restoration offers several benefits.

Direct composite restorations provide immediate feedback to the dentist during the shaping and layering process, enabling precise control over the restoration's contour, occlusion, and aesthetics, and ensuring a natural appearance that blends seamlessly with the surrounding dentition. The ability to adjust the composite material's color and translucency in real-time is particularly advantageous in achieving optimal aesthetic outcomes, vital for anterior teeth [8]. Siddiqui et al. sought to evaluate the efficacy of two methods for direct composite repair of simple crown fractures in permanent anterior teeth: Putty Index and Custom Template; 100 teeth in total were randomized into two groups - Group II (Custom Template, n=51) and Group I (Putty Index, n = 49). After the installation of the composite, both groups went through the usual processes of polishing and finishing. Blinded assessors used the modified US Public Health Service (USPHS) criteria to assess restoration quality at baseline, and six and 12 months. Spectrophotometric analysis was also used to evaluate color stability over time. A statistical study showed no noteworthy variations. But compared to Group I, Group II needed a lot more chairside time. At six and 12 months, spectrophotometric analysis showed no discernible color changes in any group [9].

Despite their numerous advantages, direct composite restorations have certain downsides. Restoration requires skill and experience to achieve results when building the crown of anterior teeth using composite resin as it is a technique-sensitive procedure. Furthermore, for larger restorations and those with high occlusal loads, direct restorations may not give the same strength and longevity as natural teeth [10]. In contrast, indirect composite restorations are made outside the oral cavity and need a multi-step approach, that begins with tooth preparation and finishes with the composite repair which was sculpted and fine-tuned with a stone model before final placement. The indirect technique offers a lot of significant benefits. Under optimal conditions, the dentist or technician can meticulously shape and detail the composite material while making the restoration outside of the mouth, making it simpler to accurately sculpt subtle anatomical features like mamelons and dentinal lobes, which are required for making natural-looking anterior restorations. Furthermore, indirect restorations may be employed in more complicated and complete settings since the composite material's strength and durability can be increased by curing it in a controlled laboratory environment [11].

Composites provide the potential to construct restorations that meet the demands and expectations of both the patient and the clinician while also appearing natural. These restorations are the result of combining the appropriate instruments and techniques. Unfortunately, certain composite insertion procedures are overlooked by dentists due to their labor-intensive nature and expertise requirements. Many tactics have been reported in the literature to help with placement and reduce chair time, such as injectable matrices, stock matrices, digitally generated matrices, and free-handing. Selecting the most appropriate approach for every individual clinical situation may facilitate and expedite the procedure. Every strategy has advantages and disadvantages, and no method is infallible or always successful. The practitioner's skill is the main criterion for selecting a case for each technique [12].

## Conclusions

Composite restorations, whether direct or indirect, provide effective treatment for dental injuries such as Ellis Class 4 fractures. Direct restorations are most effective when it comes to cautious dental preparation, chairside modifications, and efficiency. They are especially helpful in situations where maintaining the natural structure of the teeth and getting aesthetic results in a single visit are top concerns. In contrast, indirect restorations allow better control over the manipulation and shape of the material, resulting in improved strength and durability when the curing circumstances are just right. They are chosen in more complicated instances that call for detailed anatomical details or substantial repairs. The choice between direct and indirect composite restorations ultimately comes down to several variables, including the degree of tooth damage, the patient's preferences, and the clinician's level of experience. Both strategies, when used expertly and with precision, can improve patient satisfaction, restore oral health, and produce excellent functional and aesthetic results.
